# ZnS and Reduced Graphene Oxide Nanocomposite-Based Non-Enzymatic Biosensor for the Photoelectrochemical Detection of Uric Acid

**DOI:** 10.3390/bios14100488

**Published:** 2024-10-08

**Authors:** Yao Zhao, Niancai Peng, Weizhuo Gao, Fei Hu, Chuanyu Zhang, Xueyong Wei

**Affiliations:** State Key Laboratory for Manufacturing Systems Engineering, Xi’an Jiaotong University, Xi’an 710054, China; zhaoyaoxjtu@163.com (Y.Z.); pnc@mail.xjtu.edu.cn (N.P.); gaoweizhuo@stu.xjtu.edu.cn (W.G.); hufei0701@xjtu.edu.cn (F.H.); chuanyu.zhang@xjtu.edu.cn (C.Z.)

**Keywords:** biosensor, reduced graphene oxide, ZnS, uric acid, photoelectrochemical

## Abstract

In this work, we report a study of a zinc sulfide (ZnS) nanocrystal and reduced graphene oxide (RGO) nanocomposite-based non-enzymatic uric acid biosensor. ZnS nanocrystals with different morphologies were synthesized through a hydrothermal method, and both pure nanocrystals and related ZnS/RGO were characterized with SEM, XRD and an absorption spectrum and resistance test. It was found that compared to ZnS nanoparticles, the ZnS nanoflakes had stronger UV light absorption ability at the wavelength of 280 nm of UV light. The RGO significantly enhanced the electron transfer efficiency of the ZnS nanoflakes, which further led to a better photoelectrochemical property of the ZnS/RGO nanocomposites. The ZnS nanoflake/RGO nanocomposite-based biosensor showed an excellent uric acid detecting sensitivity of 534.5 μA·cm^−2^·mM^−1^ in the linear range of 0.01 to 2 mM and a detection limit of 0.048 μM. These results will help to improve non-enzymatic biosensor properties for the rapid and accurate clinical detection of uric acid.

## 1. Introduction

As one of the primary end products of purine derivatives, uric acid (UA) plays an important role in the human metabolism. UA mainly exists in urine and serum at a concentration range from 149 to 416 μM for healthy people [1]. Concentration changes in UA may indicate diseases in the metabolic system, like gout [2]. To understand the relation between clinical performance and UA concentration, a fast and accurate method for UA detection in a wide range is necessary. For uric acid measurement, frequent methods include fluorescent spectrometry [3,4,5,6,7], spectrophotometric methods [8,9,10,11], Raman spectroscopy [12], chromatography [13,14,15] and electrochemical methods [16,17,18,19]. Compared with other methods, uric acid measurement by electrochemical electrodes has advantages such as no need for expensive instruments, freedom from complex pretreatment, low cost, easy integration with flexible substrates and simple signal processing. All these make electrochemical uric acid detection more suitable for the clinical diagnosing of gout and hyperuricemia, point-of-care testing and water monitoring [20,21,22,23]. According to the difference of catalysts, electrochemical methods can be divided into enzymatic and non-enzymatic ones. Compared with the widely studied and used enzymatic method [24,25], the non-enzymatic electrochemical method does not need expensive enzymes or a complicated enzyme mobilization process. Additionally, the non-enzymatic electrochemical biosensor was much easier for storage than the enzyme-based one. To further improve the performance of the non-enzymatic electrochemical biosensor, methods like doping with high conducting elements [26] and light irradiation [27] were studied to improve electron transfer efficiency.

ZnS nanocrystals, for which photocurrent properties with UV light irradiation were reported in prior works, have been applied in hydrogen production [28] and photoelectrochemical (PEC) biosensing [29,30]. However, the productivity of hydrogen and sensitivity of PEC biosensors are restricted by the PEC response of ZnS [31,32,33]. Therefore, it is important to enhance the electron transfer efficiency of ZnS nanocrystals during PEC processing.

Graphene is a kind of two-dimensional carbon material with excellent properties and regularly serves as an electrode surface modification material [34,35,36,37,38] for the electrochemical detection of various targets. Sun et al. reported a kind of uric acid biosensor based on reduced graphene oxide (RGO) [39]. The detection results showed a wide detection range but a relatively low detection limit. RGO also cooperates with other nanomaterials to improve conducting and other properties. However, RGO in ZnS/RGO nanocomposites has not been well-studied regarding whether and how much it can enhance the electron transfer efficiency of ZnS nanocrystals to improve the PEC-detecting property.

In this work, we report a study of a ZnS/RGO nanocomposite with a better PEC property and have developed a related non-enzymatic PEC sensor for uric acid detection. It can be widely used in the clinical detection of uric acid for the early diagnosis of diseases.

## 2. Materials and Methods

### 2.1. Materials

Zinc acetate (Zn(CH_3_COOH)_2_, 99.99%), ethylenediamine (C_2_H_8_N_2_, 99%), thiourea (CH_4_N_2_S, 99.5%), terpineol (99%), uric acid (99%), RGO powder and artificial sweat were purchased from Alading (Shanghai, China).

### 2.2. Synthesis of the ZnS/RGO and Related ZnS/RGO/ITO Electrodes

ZnS nanocrystals and ZnS/RGO nanocomposites were synthesized according to previous work [40], with modification. Zinc acetate and thiourea were used as raw materials and dissolved in a mixture of water and ethylenediamine with volume ratios of 1:0 and 1:3. For the ZnS/RGO nanocomposites, 5 mg RGO powder was also added into the precursor. The mixture was heated in a Teflon autoclave and heated at 120 °C for 12 h. Then the product was washed and dried in a vacuum. Finally, ZnS/RGO nanocomposites with different morphologies were obtained.

A total of 0.05 g prepared ZnS/RGO nanocomposites were re-mixed with 0.3 mL terpineol. The mixture was then dropped on the conductive surface of a 0.25 cm × 1 cm ITO glass. The glass was put on a spin-coater and spin-coated for 1 min. After that, the modified ITO glass was heated for 8 h to remove the terpineol. Finally, the ZnS/RGO nanocomposite-covered ITO (ZnS/RGO/ITO) working electrode was acquired.

### 2.3. Characterization of the ZnS/RGO Nanocomposites

The morphology and crystal properties were characterized with field emission scanning electron microscopy (FESEM; Su-8010, Hitachi, Japan) and X-ray diffraction (XRD; D8 advance, Bruker, German). Absorption spectrums were tested with a UV-visible spectrophotometer (UV3600, Shimadzu, Japan) with a slit width of 20nm and a wavelength range of 200 to 800 nm. The lateral and longitudinal resistance of the electrodes were detected by a four-probe resistance tester. The average of ten results in the modified electrode area was taken. The photocurrent response was tested with a three-electrode electrochemical system in PBS solution with 280 nm on-and-off UV light irradiation.

### 2.4. Photoelectrochemical Test of the ZnS/RGO/ITO Working Electrodes and the Detection of Uric Acid

Before the electrochemical experiment, the ITO electrode was first rinsed with PBS and then dried by N_2_ gas. The electrochemical performance of the ZnS/RGO nanocomposite-enhanced electrode was tested with a three-electrode system and an electrochemical workstation. The PEC detection of uric acid was carried out with UV light irradiation at 280 nm. The CV results were tested at a scanning speed of 50 mV/s in the range of −0.2 V to 0.8 V in the PBS solution. I–t curves were tested by increasing the UA concentration in the PBS solution at an applied voltage of 0.4 V. Real sample detection of the UA was carried out in the artificial sweat solution in the same conditions. 

## 3. Results and Discussion

### 3.1. Characterization of ZnS/RGO Nanocomposite Electrode

**Figure 1:**
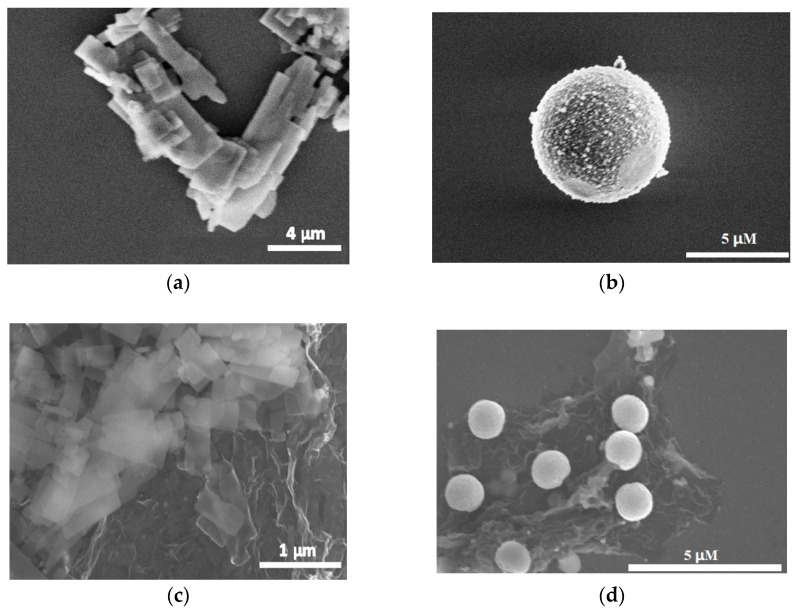
SEM images of (**a**) ZnS nanoflakes, (**b**) ZnS microparticles, (**c**) ZnS nanoflakes/RGO and (**d**) ZnS microparticles/RGO.

SEM images of the hydrothermal synthesized ZnS nanocrystals and ZnS/RGO nanocomposites are shown in Figure 1. Figure 1a,b show that at different ratios of water and ethylenediamine in the synthesis process, the ZnS nanocrystals displayed nanoflakes (H_2_O:EN = 1:3) and microparticles (H_2_O:EN = 1:0), respectively. The ZnS nanoflakes were formed because the growth of the ZnS nuclei along the [0 0 1] direction was greatly held back in the presence of ethylenediamine, whilst the growth along the direction perpendicular to the [0 0 1] direction was enhanced [41]. The diameters of the ZnS nanoparticles were 2 μm on average. The sizes of the nanoflakes were a few micrometers and the thickness a few nanometers. Figure 1c,d exhibit that for both the ZnS nanoflake/RGO and ZnS nanoparticle/RGO composites, the RGO nanoflakes were most often placed between the ZnS nanocrystals and the conductive surface of the ITO glass. Additionally, compared to the ZnS nanoparticles, the ZnS nanoflakes were wrapped more completely in the RGO nanoflakes. This suggests that the ZnS nanoflake/RGO nanocomposite had a larger contacting surface area between the ZnS and RGO than the ZnS nanoparticle/RGO one.

**Figure 2:**
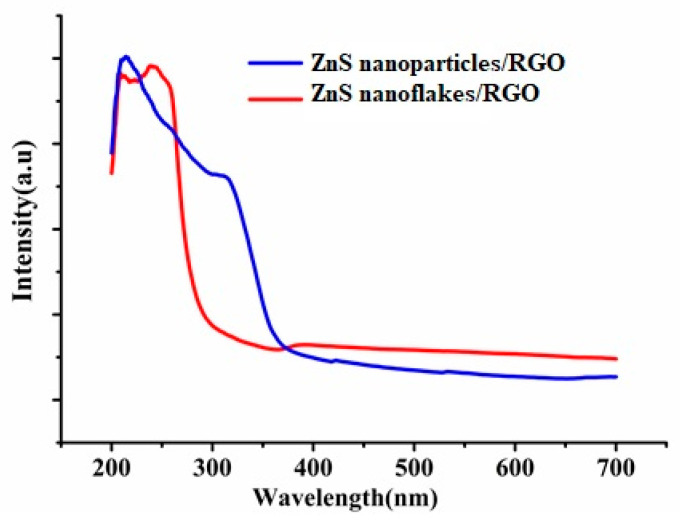
Absorption spectra of ZnS/RGO nanocomposites.

Figure 2 displays that both ZnS/RGO nanocomposites had strong absorption behavior at the region of the UV light. More importantly, at the wavelength of 280 nm, the absorption intensity of the ZnS nanoflake/RGO nanocomposite is larger than that of the ZnS nanoparticle/RGO one. This means that the ZnS nanoflake/RGO nanocomposite is more suitable for UV light at a wavelength of 280 nm.

**Figure 3:**
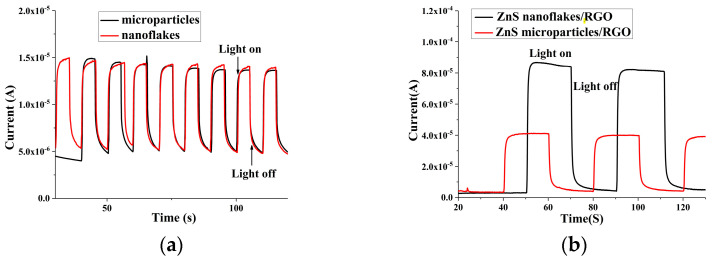

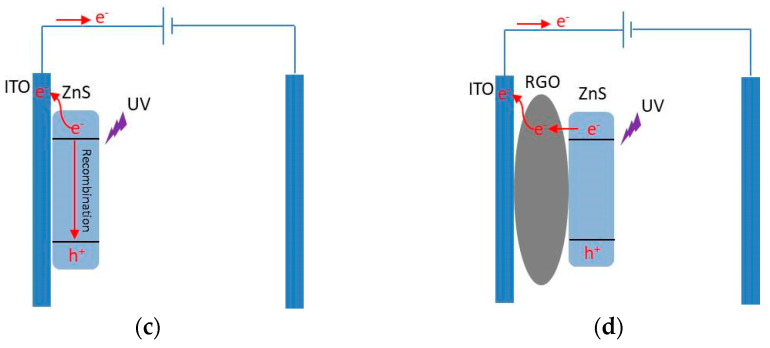
PEC responses of (**a**) ZnS nanocrystal and (**b**) ZnS/RGO nanocomposites. Schematic diagrams of the PEC responses of the (**c**) ZnS nanocrystal and (**d**) ZnS/RGO nanocomposites.

The photocurrent properties of the ZnS and ZnS–RGO nanocomposites were characterized with an irradiation of 280 nm UV light and are shown in Figure 3. Figure 3a shows that with and without the irradiation of UV light, both the ZnS microparticles and ZnS nanoflakes showed remarkable photocurrent response changes. For both ZnS nanocrystals, the ratios of I/_light_ to I/_dark_ were almost similar and counted to be 3. This is attributed to how UV light triggers the production of photoelectron–hole pairs in ZnS nanocrystals and the generated photoelectrons are transferred from the ZnS to the ITO, which finally leads to the photocurrent response.

Figure 3b exhibits that with the RGO introduced, the ratios of I/_light_ to I/_dark_ increased remarkably and reached 18.4 and 10.5 for the ZnS nanoflake/RGO and ZnS nanoparticle/RGO nanocomposites, respectively. This is because RGO, with an excellent conductivity, can trap photoelectrons and effectively inhibit the rapid recombination of generated electron–hole pairs [42]; refer to Figure 3c,d. Moreover, Figure 3b also exhibits that the I/_light_ of the ZnS nanoflakes/RGO is remarkably stronger than that of the ZnS nanoparticles/RGO. This is because compared to the latter, the former had a larger contacting surface area between the ZnS and RGO due to the more adequate encapsulation of ZnS nanocrystals with RGO nanoflakes; refer to Figure 1c,d. This made the electrons generated in the ZnS nanoflakes more easily trapped in the RGO, which further led to a better photoelectric response of the ZnS nanoflake/RGO nanocomposites.

**Figure 4:**
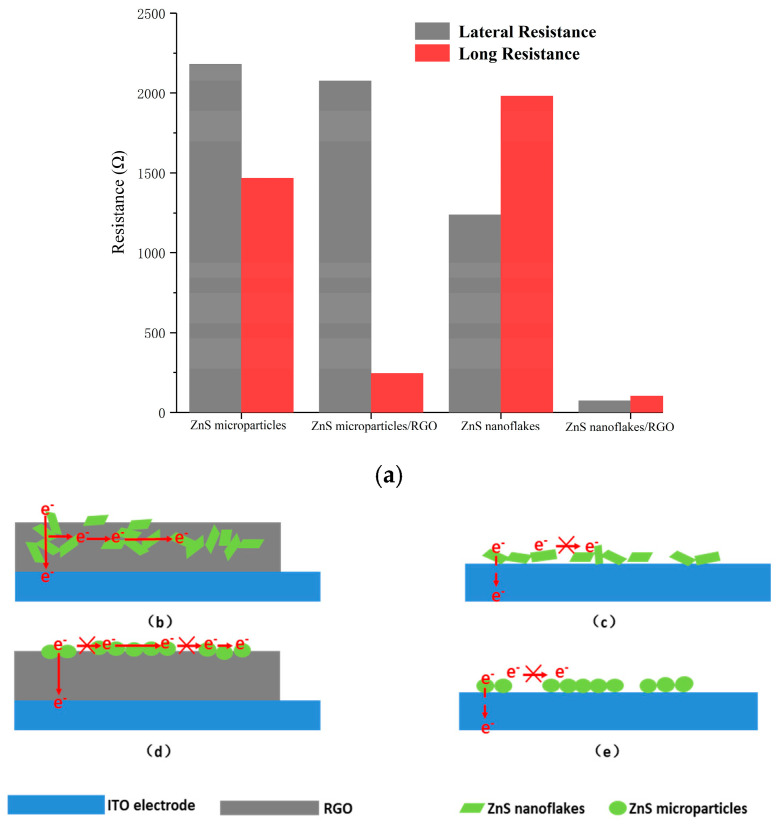
(**a**) Resistance of ZnS nanocrystals and ZnS/RGO nanocomposites along the lateral and longitudinal directions. Schematic diagrams of lateral and longitudinal electron transfer ability of the (**b**,**c**) ZnS/RGO nanocomposites and (**d**,**e**) ZnS nanocrystals.

The resistance test results for the ZnS and ZnS/RGO nanocomposite-modified electrodes are shown in Figure 4a. The lateral and longitudinal resistance test results of the ZnS nanoparticle electrode are 2180 Ω and 1467 Ω, respectively. The lateral and longitudinal resistances of the ZnS nanoparticle/RGO electrode are 2076 Ω and 244 Ω. The lateral resistance shows almost no significant change, while the longitudinal resistance has decreased to 1/6 of that of the pure ZnS nanoparticle electrode. The lateral and longitudinal resistance test results of the ZnS nanoflake electrode are 1239 Ω and 1980 Ω, while the resistances of the ZnS nanoflake/RGO electrodes are 72 Ω and 104 Ω, reduced by 1/17 and 1/19 compared to the pure ZnS nanocrystals. The encapsulation state of the ZnS nanoflakes and the RGO had significant reduction in both lateral and longitudinal resistance compared to the pure ZnS nanoflakes (Figure 4b,c), while almost all the ZnS nanoparticles were laid on top of the RGO layer, resulting in only a slight decrease in longitudinal resistance and no significant change in transverse resistance between the zinc sulfide microspheres (Figure 4c,d).

**Figure 5:**
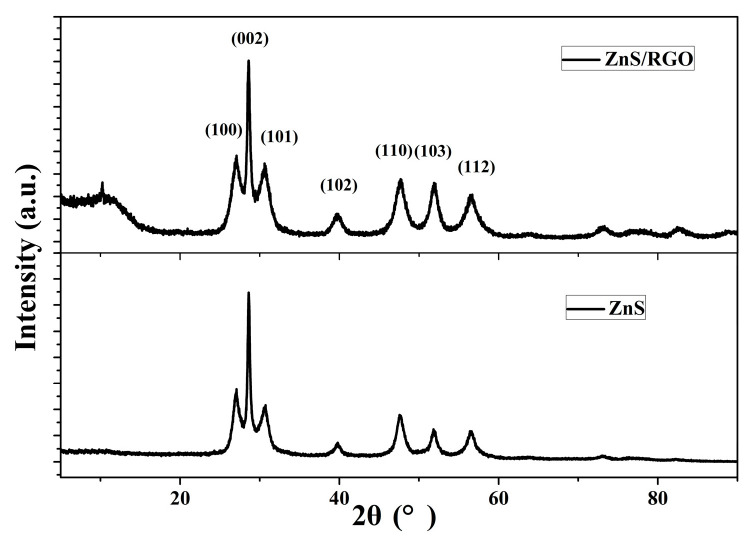
X-ray diffraction (XRD) pattern of ZnS/RGO nanocomposite and ZnS nanoflakes.

The XRD results for the ZnS nanoflakes and ZnS nanoflakes/RGO are shown in Figure 5. The peaks of 2*θ* at 27.083°, 28.617°, 30.600°, 39.802°, 47.674°, 52.050° and 56.693° of both indicate wurtzite ZnS (JCPDS File No. 75-1534). The weak peak of ZnS/RGO at 10.234° was caused by the RGO [17]. The peak of the ZnS/RGO nanocomposite is more even than that of the pure ZnS nanoflakes. This is because, differently from the pure ZnS nanoflakes, which mostly lie on the electrode, the ZnS nanoflakes are wrapped in the RGO layers (see Figure 1c), which leads to more positions and directions of the nanoflakes facing the X-ray irradiation. This suggests once more that the ZnS nanoflakes were completely encapsulated by the RGO.

**Figure 6:**
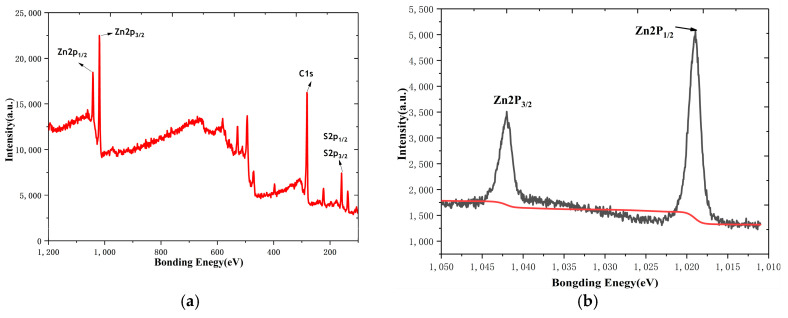

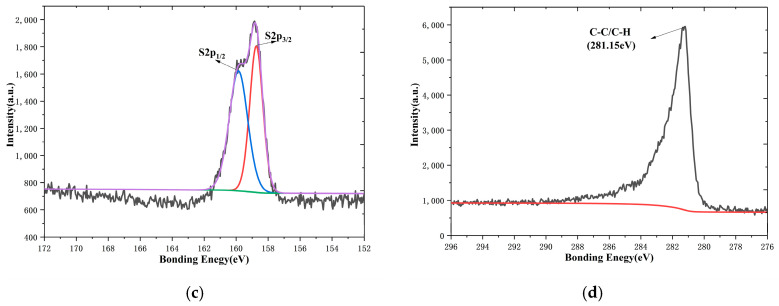
(**a**) Full XPS spectrum of ZnS nanoflakes/RGO, (**b**) Zn2p XPS spectrum, (**c**) S2p XPS spectrum and (**d**) C1s XPS spectrum.

Figure 6a shows the full spectrum of the ZnS nanoflakes/RGO, in which the C1s peak; Zn2p_1/2_ and Zn2p_3/2_ peaks; and S2p_1/2_ and S2p_3/2_ peaks can be found. This indicates the existence of a RGO and zinc sulfide compound. The XPS spectrum of Zn2p in Figure 6b has two main peaks, at 1018.97 eV and 1041.09 eV, corresponding to 2p_1/2_ and 2p_3/2_, respectively, indicating Zn^2+^ in a normal oxidation state [42]. Figure 6c shows the S2p_3/2_ and S2p_1/2_ peaks for S^2−^ at 158.75 eV and 160.05 eV [42]. The peak at 281.15 eV of the spectrum of C1 in Figure 6d can be attributed to the presence of the C-C bond of the RGO [42].

### 3.2. Electrochemical Properties and Photoelecrochemical Detection of Uric Acid by the ZnS Nanoflake/RGO Electrode

**Figure 7:**
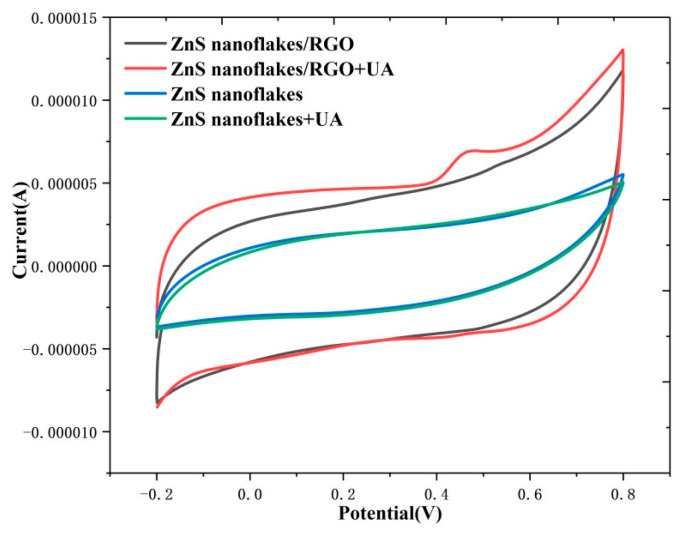
Cyclic voltammetry curves of ZnS and ZnS/RGO on the ITO electrode with 0.1 mM UA and without UA at a scanning speed of 0.05 V.

Cyclic voltammetry (CV) can reflect the redox reaction on the surface of a working electrode well. Figure 7 shows that compared to pure ZnS nanoflakes, the ZnS nanoflakes/RGO show a larger current at voltage from −0.2 to 0.8 V. This is due to the better conductivity of the ZnS nanoflakes/RGO than that of the pure ZnS nanoflakes, which is in agreement with the results of Figure 4. More importantly, with and without uric acid added, only the CV curve of the ZnS nanoflakes /RGO shows a clear peak at 0.4 V. This indicates that, attributable to the better conductivity and photocurrent behavior of the ZnS nanoflakes/RGO, the ZnS nanoflake/RGO nanocomposite had better electrocatalytic activity than the pure ZnS nanoflakes; refer to Figure 3 and Figure 4.

**Figure 8:**
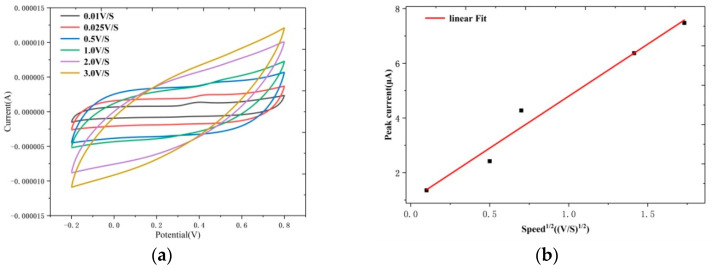
(**a**) Cyclic voltammetry curves of ZnS nanoflakes/RGO with UA at different voltage scanning speeds and (**b**) the linear fit curve of the current peak versus the square root of the scanning rate.

The effect of the scanning rate on the oxidation of UA on the ZnS nanoflake/RGO/ITO electrode was also studied. Figure 8a shows that with the scanning rate increasing, the peak current at 0.4 V increases and presents a small right shift. Based on Figure 8a, the linear correlation of the current peak versus the square root of the scanning rate was determined; refer to Figure 8b. The linear equation was defined as I(μA) = 3.798 V^1/2^(V/s) + 0.999, R^2^ = 0.9678. This means that the surface reaction on the ZnS nanoflake/RGO electrode is a diffusion-controlled process [43,44]. That is, the faster the diffusion speed between the redox sites of the ZnS nanoflakes/RGO and UA is, the stronger the UA oxidation on the ZnS nanoflakes/RGO is. This further suggests that there exist sufficient redox sites on the ZnS nanoflakes/RGO for the uric acid redox reaction due to the excellent PEC performance of the ZnS nanoflakes/RGO.

**Figure 9:**
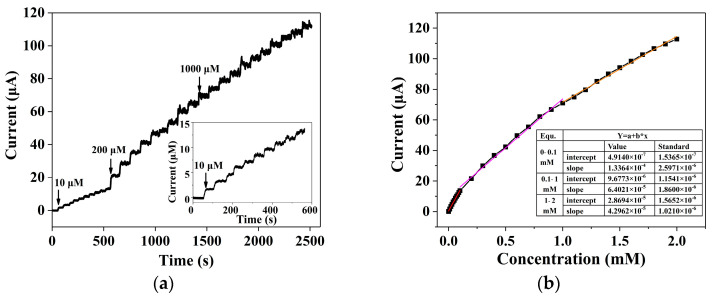
I–t response (**a**) and linear calibration (**b**) of the ZnS/RGO working electrode with continuous addition of 2 mM uric acid at an applied potential of 0.4 V. The inset is the i–t response with 0–0.1mM uric acid.

The PEC amperometric response of the ZnS nanoflake/RGO/ITO electrode with UA is shown in Figure 9a. The current rises quickly as the UA concentration increases from 0.01 to 2 mM. The linear calibration curves of the current response in Figure 9b show different sensitivities in three linear ranges. The equations of linear fitting were calculated to be y = 1.336e^−4^ x + 4.9148e^−7^ at the range of 0–0.1 mM (R^2^ = 0.9962), y = 6.402e^−5^ x + 9.677e^−6^ at the range of 0.1–1 mM (R^2^ = 0.9924) and y = 4.296e^−5^ x + 2.869e^−5^ at the range of 1–2 mM (R^2^ = 0.9944). Considering the reaction area of 0.25 cm^2^, the sensitivities were 534.5 μA·cm^−2^·mM^−1^, 256.1 μA·cm^−2^·mM^−1^ and 171.9 μA·cm^−2^·mM^−1^, respectively, which are higher than that of the ZnS enzyme-based uric acid biosensor in our previous work [40] and other uric acid biosensors listed in Table 1 [45,46,47,48,49]. The decrease in sensitivity with the higher UA concentration was due to the saturation of catalytic ability [50,51]. That is, with the UA concentration increasing, reaction products and unreacted UA species gradually foul the surface of ZnS nanoflakes/RGO, which leads to the inevitable reduction in active sites and conductivity [50,51]. Conversely, thanks to the protection of RGO, some active sites on the ZnS nanoflakes/RGO for UA oxidation still exist, giving rise to the considerable linear range of 0.01–2 mM of the electrode. The detection limit of this uric acid sensor was 0.048 μM, obtained by a signal-to-noise ratio (S/N = 3) method. The sensitivity was higher than that of most enzymatic electrochemical methods. Compared to other non-enzymatic methods, the linear range of 0.01–2 mM is suitable to cover the concentrations of UA in 0.1–0.5 mM urea and 0.01–0.05 mM sweat of a healthy human. All this shows that the ZnS/RGO nanocomposite-based non-enzymatic biosensor in this paper is suitable for the rapid and accurate clinical detection of uric acid in multiple human samples.

### 3.3. Anti-Interference Capability, Long-Term Stability and UA Detection in Real Samples

**Figure 10:**
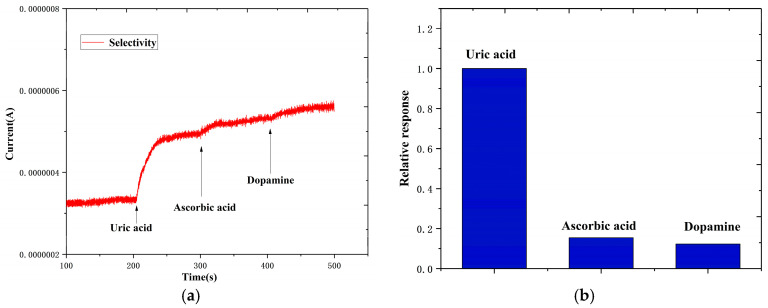
(**a**) Current responses of ZnS nanoflakes/RGO with 10 μM uric acid, 10 μM ascorbic acid and 10 μM dopamine. (**b**) Relative responses of uric acid, ascorbic acid and dopamine.

Figure 10a shows that compared to the increased current caused by uric acid, the current responses of dopamine and ascorbic acid were significantly small. The relative responses of the ascorbic acid and dopamine compared with the uric acid are 0.1543 and 0.1234, respectively; refer to Figure 10b.

These results suggest that the electrochemical electrode shows excellent selectivity for uric acid measurement. This is due to the chosen oxidization potential of 0.4 V and the sufficient redox sites provided by the ZnS nanoflake/RGO electrode. At the oxidization potential of 0.4 V, the current response of the UA was much higher than those of the DA and AA on the carbon-based electrode [52]. Additionally, the sufficient redox sites significantly promoted the UA response of the ZnS nanoflake/RGO electrode. All this is attributed to the excellent selectivity of the electrochemical electrode for uric acid measurement.

**Figure 11:**
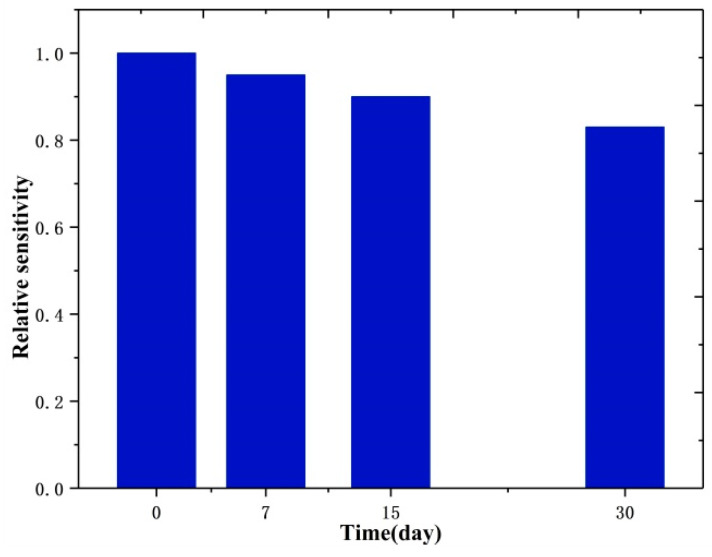
Long-term stability of the ZnS nanoflakes/RGO with uric acid at 0, 7, 15 and 30 days during one month.

The signal change during long-term usage for the UA biosensor has been determined and is shown in Figure 11. It was observed that the original response of the ZnS nanoflake/RGO electrode with UA remained at about 90% until 15 days later and 83% after a month, which is larger than those of the non-enzymatic and enzymatic electrochemical sensors (80% and 67.4% after 30 days) in previous works [53,54]. This is ascribed to the RGO, which efficiently protects the active sites on ZnS nanoflakes/RGO and provides the friendly microenvironment with an inertia property. This suggests that the PEC electrode has great long-term stability. In addition, the reduction (around 17% after 30 days) in sensitivity was mainly caused by the active site consumption of the electrode in the air.

**Figure 12:**
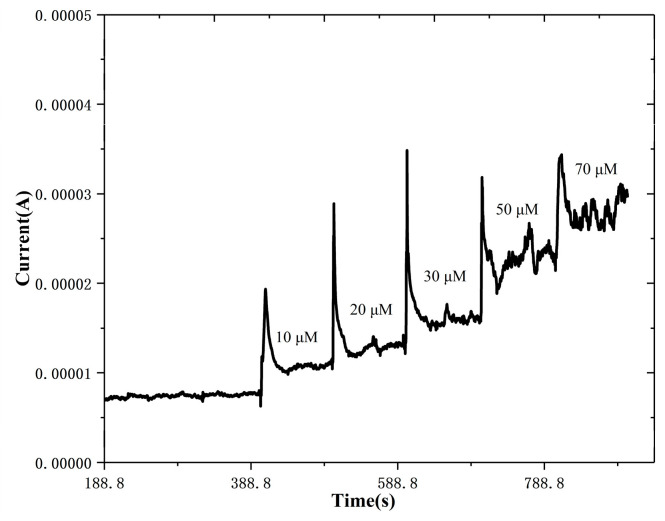
Current response of the ZnS/RGO working electrode with continuous addition of uric acid at an applied potential of 0.4 V in artificial sweat.

Figure 12 exhibits that there exists a remarkable current response of the ZnS nanoflake/RGO biosensor with the UA added into the artificial sweat solution. The UA concentration recovery of the determination by the biosensor to the addition was from 85.1% to 105%, as in Table 2. This indicates that ZnS nanoflakes/RGO can be employed for UA detection in human sweat.

## 4. Conclusions

In this work, ZnS/RGO nanocomposites were synthesized by using the hydrothermal method. The nanomaterial on ITO glass was fabricated as a non-enzyme PEC biosensor for uric acid sensing. The ZnS nanoflakes/RGO exhibited a higher PEC property, with 280 nm UV light irradiation, due to the better conductivity of the RGO. The uric acid detection results showed a highest sensitivity of 534.5 μA·cm^−2^·mM^−1^ in the range of 0.01 mM to 2 mM, with a minimum sensitivity of 171.9 μA·cm^−2^·mM^−1^ and a detection limit of 0.048 μM, which meets the requirement for clinical applications. Finally, quantitative determination of UA was successfully performed in artificial sweat. These results demonstrate that the high catalytic and conducting properties of the ZnS/RGO nanocomposite make it a promising nanomaterial for non-enzymatic PEC biosensing applications.

## Figures and Tables

**Table 1:** Comparison of ZnS nanoflake/RGO/ITO PEC detection of uric acid with other methods. Units of sensitivity, LOD and linear range are μA·cm^−2^·mM^−1^, μM and mM.

Materials	Method	Sensitivity	LOD	Linear Range	Reference
Nafion/uricase/ZnS nanoparticles/ITO	Enzymatic electrochemistry	43.18	1.79	0.01–1.5	[40]
Nafion/uricase/ZnS urchin-like nanostructures/ITO	Enzymatic electrochemistry	76.12	0.70	0.01–1.7	[40]
Nafion/uricase/ZnS nanoflakes/ITO	Enzymatic electrochemistry	34.28	1.51	0.01–2.0	[40]
Nafion/uricase/ZnO–RGO/ITO	Enzymatic photoelectrochemistry	427.8	0.039	0.01–0.2	[45]
Nafon/uricase/ZnO–RGO-Au/ITO	Enzymatic electrochemistry	768.6	0.29	0.2–2	[46]
RGO/PEDOT:PSS/GCE	Non-enzymatic electrochemistry		0.05	0.01–0.1	[47]
Co-N/C@MWCNTs	Non-enzymatic electrochemistry		0.09	0.001–0.04	[48]
ZnO film/RGO/ITO	Non-enzymatic electrochemistry	150.7	0.45	1–40	[49]
ZnS nanoflakes–RGO/ITO	Non-enzymatic photoelectrochemistry	534.56	0.048	0.01–2	This work

**Table 2:** PEC detection and recovery results of UA in artificial sweat with ZnS nanoflakes/RGO by the standard addition method.

Sample	Added (μM)	Found (μM)	Recovery (%)
1	10	10.50	105
2	10	8.51	85.1
3	10	10.10	101.0
4	20	17.32	85.16
5	20	17.56	87.80

## Data Availability

Data will be made available on request.
